# Supplementary material for: Effect of Spherical Adsorptive Carbon Among Chronic Kidney Disease Patients: A Nationwide Cohort Study
Source: Int J Environ Res Public Health. 2025 Aug 30;22(9):1365. doi: 10.3390/ijerph22091365 (PMC12470180; doi:10.3390/ijerph22091365)
Supplement: Supplementary file 1 [file ijerph-22-01365-s001.zip › ijerph-3750408-supplementary.pdf]

Supplementary Materials

Table S1. Diagnosis, procedure, and specific codes.

Disorder	Diagnosis, Procedure, Specific Codes
ESKD	N18.5, N18.6
Dialysis	Z49.1, Z49.2
Acquired immunodeficiency syndrome	B20 B21, B22, B24
Cancer	C00 to C97
Cerebrovascular disease	G45, G46, I60, I61, I62, I63, I64, I65, I66, I67, I68, I69, H340
Chronic lung disease	J40, J41, J42, J43, J44, J45, J46, J47, J60, J61, J62, J63, J64, J65, J66, J67, I278, I279, J684, J701, J703
Diabetes	E10, E11, E12, E13, E14
Dyslipidemia	E780
Hematologic malignancy	C00 to C97 (except C77, C78, C79, C80)
Hemiplegia	G81, G82, G041, G114, G801, G802, G830, G831, G832, G833, G834, G839
Hypertension	I10, I11
Kidney disease	N183, N184, N185, N186
Kidney transplantation	Z94.0, T86.1
Metastatic solid tumor	C77, C78, C79, C80
Mild liver disease	B18, K73, K74, K700, K701, K702, K703, K709, K713, K714, K715, K717, K760, K762 K763, K764, K768, K769, Z944
Moderate to severe liver disease	I850, I859,I864, I982, K704, K711, K721, K729, K765, K766, K767, K704, K711
Peripheral artery disease	I700 to I702, I708, I709, K551, K558, K559, Z958, Z959, I1731, I1738, I1739, I1771, I1790, I1792
Rheumatologic disease	M05, M06, M32, M33, M34, M315, M351, M353, M360
Stroke	I60 to I62 (hemorrhagic stroke), I63 (ischemic stroke)

All diagnostic, procedure, and specific codes are shown at https://www.hira.or.kr/.

Table S2. Drug codes used for National Health Insurance Korea database analysis.

Drug	Health Insurance Review & Assessment (HIRA) Service Code
ACEi or ARBs or aliskiren	104201ATB, 104202ATB, 114701ATB, 122601ATB, 122602ATB, 122603ATB, 122901ATB, 122902ATB, 122903ATB, 133001ATB, 133002ATB, 133003ATB, 140901ATB, 140902ATB, 151601ATB, 151603ATB, 163501ATB, 163502ATB, 104201ATB, 104202ATB,114701ATB, 122601ATB, 122602ATB, 122603ATB, 122901ATB, 122902ATB, 122903ATB, 133001ATB, 133002ATB, 133003ATB, 140901ATB, 140902ATB, 151601ATB, 151603ATB, 163501ATB, 163502ATB, 173401ATB, 173402ATB, 177301ATB, 177303ATB, , 177303ATB, 184501ATB, 185701ATB, 185702ATB, 196801ATB, 196802ATB, 211301ATB, 211302ATB, 221901ATB, 222401ACH, 222401ATB, 222402ACH, 222402ATB, 222404ATB, 235002ATB, 247101ATB, 247102ATB, 247103ATB, 247104ATB, 378801ATB, 378802ATB, 378803ATB, 429201ATB, 468501ATB, 468502ATB, 468503ATB, 501601ATB, 501602ATB, 510401ATB, 510402ATB, 510403ATB, 515201ATB, 515202ATB, 515203ATB, 520901ATB, 520902ATB, 662401ATB, 662402ATB, 662403ATB, 651401ATB, 651402ATB, 651403ATB
BBs	117903ATB, 117904ATB, 124801ATB, 219901ATB, 219902BIJ, 219904ATB, 219905ACR, 219906ACR, 111401ATB, 111402ATB, 111403ATB, 116801ATB, 116803ATB, 117001ATB, 117002ATB, 125001ATB, 125002ATB, 125003ATB, 125004ACR, 125005ATB, 125006ACR, 125007ACR, 125007ATR, 125008ACR, 125008ATR, 483101ATB, 483102ATB, 489501ATB, 489502ATB, 489503ATB, 662201ATB, 662202ATB, 117901ATB, 117902ATB, 129101ATB, 193802ATB, 194003ATR, 198301ATB, 154401BIJ, 154402BIJ, 154431BIJ, 154430BIJ, 180201ATB, 180201BIJ, 180202BIJ, 180230BIJ, 180231BIJ
CCBs	107601ATB, 107601ATD, 107602ATB, 107602ATD, 107603ATB, 114001ACH, 114002ACH, 114003ACH, 115101ATB, 115102ATB, 115103ATB, 115104ATB, 133101ATB, 133102ATB, 157501ATR, 157503ATR, 178902ACR, 180301ATB, 180302ATB, 180303ATB, 182001ATB, 182002ATB, 188001ATB, 188002ATB, 201001BIJ, 201002ATB, 201002BIJ, 201003ACR, 201030BIJ, 201031BIJ, 201033BIJ, 202401ATB, 202402ACS, 202402ATB, 247601ACR, 247603ATR, 247604BIJ, 247605ATR, 247606ATB, 247607ATB, 459801ACH, 459801ATB, 459802ACH, 459901ATB, 459902ATB, 464601ATB, 470801ATB, 470802ATB, 476201ATB, 479701ATB, 483201ATB, 483202ATB, 489501ATB, 489502ATB, 489503ATB, 145702BIJ, 145703ACR, 145704BIJ, 145706ATB, 145706ATR, 145707ACR, 145707ATB, 145707ATR, 201401ACS, 201401ATB, 201402ATB, 201405ATR, 201407ACS, 201408ATR, 201409ATR, 201702ATB, 201901ATB, 201902BIJ, 201930BIJ, 356201ATB, 356202ATB, 356202ATR, 356203ATR, 441201ATB, 441202ATB, 528201ATR, 528202ATR, 145702BIJ, 247630BIJ, 486501ATB, 486502ATB, 495901ATB, 501701ATB
Diuretics	101501ATB, 101502BIJ, 106901ATB, 163801ATB, 163802BIJ, 163830BIJ, 170801ATB, 174401ATR, 174402ATB, 174403ATB, 231101ATB, 231102ATB, 244701ATB, 262700ATB, 367001ATB, 367002ATB, 451301ATB, 451302ATB
Vasodilators	170701ATB, 170702BIJ, 196102ATB, 471401CSI, 471430CSI, 485201ATB, 485202ATB, 553301ATB, 564701ATB, 564702ATB, 632201ATB, 652301ATB, 652302ATB, 652303ATB, 170730BIJ, 512401BIJ, 512402BIJ, 512403BIJ, 512430BIJ, 512431BIJ, 512432BIJ,
ACEi or ARBs and CCBs	447100ATB, 447200ATB, 466000ATB, 492800ATB, 492900ATB, 495800ATB, 500500ATB, 500600ATB, 582200ATB, 582400ATB, 502700ATB, 503000ATB, 513900ATB, 511500ATB, 511600ATB, 511700ATB, 623100ATB, 521200ATB, 521300ATB, 521400ATB, 644800ATB, 522200ATB, 522300ATB, 522400ATB, 522600ATB, 522700ATB, 522800ATB, 522900ATB, 523000ATB, 523100ATB, 523200ATB, 523300ATB, 523400ATB, 547500ATB, 547600ATB, 547700ATB,

	547800ATB, 547900ATB, 548000ATB, 631300ATB, 629400ATB, 629500ATB, 629600ATB, 632800ATB, 632900ATB, 633000ATB, 637400ATB, 637500ATB, 637600ATB, 644800ATB, 651900ATB, 652000ATB, 652100ATB, 652700ATB, 652900ATB, 653000ATB, 653100ATB
ACEi or ARBs and statins	524000ATB, 524100ATB, 527000ATB, 527100ATB, 525000ATB, 525100ATB, 525200ATB, 525300ATB, 629700ATB, 629800ATB, 526300ATB, 526400ATB, 526500ATB, 526900ATB, 644100ATB, 644200ATB, 653200ATB, 629900ATB, 630000ATB, 630100ATB, 630200ATB, 631600ATB, 631700ATB, 634900ATB, 635000ATB, 635100ATB, 635200ATB, 653200ATB, 654600ATB, 654700ATB, 654800ATB, 654900ATB, 655000ATB, 661800ATB, 661900ATB, 662000ATB, 662100ATB, 673700ATB, 688100ATB, 688200ATB, 688300ATB, 688400ATB, 688500ATB
BB with diuretics	262100ATB, 262600ATB, 460200ATB, 469800ATB, 469900ATB, 470000ATB
ACEi or ARBs and diuretics	262200ATB, 262300ATB, 262500ATB, 378900ATB, 440300ATB, 453600ATB, 453700ATB, 486900ATB, 356400ATB, 442600ATB, 385700ATB, 385800ATB, 423700ATB, 440800ATB, 443200ATB, 443300ATB, 502600ATB, 448600ATB, 448700ATB, 460500ATB, 477400ATB, 490100ATB, 497900ATB, 499200ATB, 499300ATB, 513600ATB, 522000ATB, 526800ATB, 556200ATB, 673500ATB, 673600ATB
ACEi or ARBs and CCBs and diuretics	519700ATB, 519800ATB, 519900ATB, 520000ATB, 520100ATB, 662800ATB, 662900ATB, 663000ATB, 663500ATB, 663600ATB, 663700ATB, 663800ATB, 682700ATB, 682800ATB, 682900ATB
ACEi or ARBs and CCBs and statins	663900ATB, 664000ATB, 664100ATB, 664200ATB, 664300ATB, 664400ATB, 671200ATB, 671300ATB, 671400ATB, 671500ATB, 671600ATB, 671700ATB, 677000ATB, 677100ATB, 677300ATB, 677400ATB, 677500ATB, 677600ATB, 686800ATB, 679500ATB, 679600ATB, 679700ATB, 680300ATB, 684300ATB, 684400ATB, 684500ATB, 684600ATB, 684700ATB, 686800ATB, 686900ATB, 690400ATB, 690500ATB, 690600ATB, 690700ATB, 691400ATB, 691500ATB
CCBs and statins	472300ATB, 472400ATB, 472500ATB, 518900ATB, 614500ATB, 673900ATB, 674000ATB, 674100ATB, 678600ATB
BBs and CCBs	262400ATR
BBs and statins	683000ATB, 683100ATB, 683200ATB, 691200ATB
Alpha blockers	149101ATB, 149102ATB, 149104ATR, 483401ACH, 104803ATR, 159001ATB, 234601ACR, 234601ATD, 234601ATR, 234602ACR, 234603ACR, 234603ATD, 234603ATR, 235501ATB, 235502ATB, 235503ATB, 458801ACS, 458801ATB, 504201ACH, 504202ACH, 504202ATB, 504203ACH, 504203ATD, 505801ATB, 505802ATD, 614201ATB, 614202ATB, 614203ATB
Cyclosporin or tacrolimus	234201ACH, 234201ATB, 234203ACH, 234203ATB, 234204ACH, 234204ATB, 234208ATB, 234230BIJ, 234205ACR, 234206ACR, 234207ACR, 139201ACS, 139204ACS, 194730ALQ, 194701ACS, 194702ACS, 139230BIJ
Statins or ezetimibe or fibrate	111501ATB, 111502ATB, 111503ATB, 111504ATB, 162401ACH, 162402ACH, 162403ATR, 185801ATB, 216601ATB, 216602ATB, 216603ATB, 216604ATB, 218001ATB, 227801ATB, 227801ATR, 227802ATB, 227803ATB, 227805ATB, 227806ATB, 454001ATB, 454002ATB, 454003ATB, 462201ATB, 470901ATB, 470902ATB, 470903ATB, 471000ATB, 471100ATB, 507800ATB, 502201ATB, 502202ATB, 502203ATB, 502204ATB, 519300ACH, 631400ATB, 631500ATB, 633800ATB, 633900ATB, 634600ATB, 634800ATB, 640700ATB, 640800ATB, 640900ATB, 663400ACS, 679300ACH
DM medications	170101BIJ, 170102BIJ, 170103BIJ, 170130BIJ, 170131BIJ, 170401BIJ, 170402BIJ, 170430BIJ, 170431BIJ, 170502BIJ, 175301BIJ, 175302BIJ, 175304BIJ, 175330BIJ, 175331BIJ, 175332BIJ, 175333BIJ, 441301BIJ, 441302BIJ, 441303BIJ, 441304BIJ, 441305BIJ, 441330BIJ, 441331BIJ, 441332BIJ, 441333BIJ, 441334BIJ, 461801BIJ, 461802BIJ, 461804BIJ, 461830BIJ, 461831BIJ, 461832BIJ, 484901BIJ, 484902BIJ,484930BIJ, 484931BIJ, 488701BIJ, 488730BIJ, 507401BIJ, 626700BIJ, 626801BIJ, 626802BIJ, 626830BIJ,

	626831BIJ, 512101BIJ, 512102BIJ, 512130BIJ, 512131BIJ, 626601BIJ, 626602BIJ, 626630BIJ, 626631BIJ, 639701BIJ, 639702BIJ, 644501BIJ, 644502BIJ, 666700BIJ, 667000BIJ, 527301ATB, 527302ATB, 628201ATB, 628202ATB, 636101ATB, 639800ATR, 641400ATR, 649000ATB, 649100ATB, 649200ATB, 649300ATB, 649400ATB, 649500ATB, 674301ATB, 674302ATB, 100601ATB, 100602ATB, 165402ATB, 165601ACS, 165602ACS, 165602ATB, 165603ATR, 165604ATR, 165701ATB, 165702ATB, 165703ATB, 165704ATB, 165801ATB, 191501ATB, 191502AGR, 191502ATB, 191502ATR, 191503ATB, 191504ATB, 191504ATR, 191505ATR, 249001ATB, 249002ATB, 249002ATD, 348002ATB, 379501ATB, 379502ATB, 379503ATB, 406201ATB, 406202ATB, 421100ATB, 430201ATB, 430202ATB, 430203ATB, 431901ATB, 431902ATB, 443400ATB, 443500ATB, 452700ATB, 452900ATB, 469100ATB, 471900ATB, 474200ATB, 474300ATB, 474300ATR, 488800ATB, 488900ATB, 489000ATB, 498100ATB, 498600ATB, 486101ATB, 497200ATB, 498100ATB, 523600ATB, 523700ATB, 525500ATB, 525600ATB, 525901ATB, 631900ATB, 632100ATB, 637200ATB, 653800ATR, 653900ATR, 654000ATR, 655700ATR, 518800ATB, 500801ATB, 501101ATB, 501102ATB, 501103ATB, 502200ATB, 502300ATB, 502300ATR, 502900ATB, 513700ATB, 513700ATR, 524700ATR, 507000ATB, 507100ATB, 519600ATB, 518500ATR, 518600ATR, 520500ATB, 520600ATB, 520700ATB, 523800ATR, 632000ATR, 645000ATR, 654100ATR, 613301ATB, 613302ATB, 616401ATB, 619101ATB, 624201ATB, 624202ATB, 624203ATB, 627301ATB, 630300ATB, 630400ATB, 630500ATB, 630600ATB, 635600ATB, 635700ATB, 675500ATB, 639601ATB, 641800ATR, 641900ATR, 642000ATR, 645301ATB, 648400ATB, 648500ATB, 648600ATB, 649900ATR, 650000ATR, 650100ATR
DM medications and statins	664600ATB, 664700ATB, 664800ATB, 671800ATR, 673800ATR, 671900ATR, 672000ATR, 672100ATR, 672500ATR, 672600ATR, 672700ATR, 672800ATR, 672900ATR, 673000ATR, 683300ATR, 683400ATR
NSAIDs	100901ACH, 100901ACS, 100901ATB, 100903ATR, 142331ASY, 142332ASY, 142333ASY, 142334ASY, 142336ASY, 142337ASY, 142301ACS, 142301ATB, 142301ATR, 142302ATB, 142303ATB, 143402ATB, 143504ATB, 143506ATB, 172833ASY, 172834ASY, 172835ASY, 172836ASY, 172837ASY, 172838ASY, 172802ACS, 172802ATB, 172806ATB, 172807ATR, 173502ATB, 180001ATB, 186101ATB, 186102ATR, 189001ACH, 189003ATB, 189701ACH, 189701ATB, 189701ATD, 189702ACH, 189702ATD, 201801ATB, 216534ASY, 218602ATB, 234401ATB, 238901ATB, 251800ATB, 251800ATB, 347701ACH, 347701ATB, 347702ACH, 347702ATB, 347703ACH, 355501ATB, 355503ATB, 458402ATB, 493401ATB, 493402ATR, 636401ACH, 636401ATB, 636901ATB, 313400ACH, 313400ACH, 313400ACH, 143534BIJ, 143535BIJ, 143630BIJ, 143631BIJ, 179731BIJ, 180033BIJ, 214031BIJ, 214130BIJ, 454330BIJ, 172830BIJ
Glucocorticoids	116401ATB, 140801ATB, 141901ATB, 141903ATB, 160201ATB, 170901ATB, 170906ATB, 193302ATB, 193305ATB, 217034ASY, 217035ASY, 217001ATB, 243201ATB, 243202ATB, 243203ATB, 296900ATB
AST-120	459701AGN, 459701ATD, 459702ACH
SGLT2 inhibitors	527301ATB, 527302ATB, 628201ATB, 628202ATB, 636101ATB, 639800ATR, 641400ATR, 649000ATB, 649100ATB, 649200ATB, 649300ATB, 649400ATB, 649500ATB, 674301ATB, 674302ATB

ACEi, angiotensin-converting enzyme inhibitors; ARB; angiotensin receptor blocker; BB, beta blocker; CCB, calcium channel blocker; DM, diabetes mellitus.
